# Effects of combined extracts of *Lepidium meyenii* and *Allium tuberosum* Rottl. on erectile dysfunction

**DOI:** 10.1186/s12906-019-2542-4

**Published:** 2019-06-18

**Authors:** Yi Zhang, Feixian Zhou, Fahuan Ge

**Affiliations:** School of Pharmaceutical Sciences, Sun Yat-Sen University, Guangzhou, 510006 China

**Keywords:** Combined extracts, Maca, Chinese chive seed, Supercritical fluid, Sexual function

## Abstract

**Background:**

Sexual problems are widespread and adversely affect the interpersonal relationships and the quality of life. Currently, synthetic drugs improving sexual function are available, but expenditures for such agents are extremely high. To discover relatively inexpensive, widely available and effective natural drugs, we identified a combined extracts from *Lepidium meyenii* (maca) root and *Allium tuberosum* Rottl. (Chinese chive) seed, assessed the effects of this combined extracts on erectile dysfunction, and explored its potential mechanisms.

**Methods:**

The extracts were obtained via supercritical fluid extraction. Male BALB/c mice received doses of extract from single plant or the combined extracts (200 mg/kg) by gastric gavage for 14 d, and Viagra was used as the positive control drug. Sexual behaviour was observed, and concentrations of serum testosterone, nitric oxide (NO), and cyclic guanosine monophosphate (cGMP) in serum as well as in penis were measured. In addition, weights of genital organs were also measured.

**Results:**

The combined extracts of maca root and Chinese chive seed (1:1, w/w) had a 45-fold increase in macamide content compared with maca extract. It also led to significantly higher ejaculation frequency (*P* < 0.05) than single extract from maca root or Chinese chive seed, with no corresponding effect on genital indices. In addition, the NO level in serum (*P* < 0.01) and penis (*P* < 0.05) increased notably, as well as the level of cGMP in penis (*P* < 0.05).

**Conclusions:**

The results indicated that the combined extracts produced better synergistic effects on male sexual function than maca extract or Chinese chive extract alone. These positive effects may involve the upregulation of NO and cGMP concentrations in penis.

## Background

Health concerns related to male sexual function are becoming a global problem due to the great pressure from economic, social relationship and physiological demands. Sexual dysfunction was defined as a heterogeneous group of disorders to respond sexually or to experience sexual pleasure [1]. Sexual dysfunction, especially erectile dysfunction (ED) that is also known as impotence, is a serious public health problem according to NIH report [2]. The increasing number of men seeking treatment for impotence has extended the basic physiological and pharmacological research on sexual performance [3]. Male sexual excitement is identified by the rise of sexual desire in the brain and the subsequent transmission of relevant signals to the periphery, followed with the penile tumescence necessary for sexual intercourse [4]. Many types of synthetic agents are available for improving sexual function, such as sildenafil, vardenafil, tadalafil, avanafil and udenafil [5–7]. However, long-term use of these drugs can produce negative side effects, such as headache [5, 8], muscle pain [8], blurred vision [8], and impairment of renal or hepatic function [9]. Moreover, these agents are expensive. Thus, the exploration of relatively inexpensive, widely available and effective natural drugs from medicinal plants that have been used in alternative therapies is of great significance on global health, particularly for developing countries [10].

*Lepidium meyenii*, which is also known as maca, has been cultivated for thousands of years as a food supplement and a traditional medicine [11]. Several studies have shown that maca possesses many bioactivities, such as enhancing sexual function [12–16], increasing fertility [17] and improving sperm parameters [18–23]. In addition, macamides and macaene are representative marker compounds for quality control of maca [24, 25] due to their biologically activities [26]. The seeds of Chinese chive (*Allium tuberosum* Rottl.) have been used in traditional Chinese medicine for treating impotence and nocturnal emissions [27]. Meanwhile, Chinese chives have been widely cultivated in China and used not only as a foodstuff but also in folkloric medicine. This plant is one of the edible green vegetables consumed daily by Chinese. It has been known that plants which possess the same properties can be combined in order to enhance their biological activities [10, 28, 29]. Several studies have reported that maca extract and the seed of Chinese chive enhanced the sexual function by increasing the number of intromissions [26] or reducing intromission latency [30]. However, there was no experimental investigation performed on the sexual improvement effects of combined extracts from maca and Chinese chive seed.

In the present study, we investigated the effects of single extract from maca root or Chinese chive seed and the combined extracts from these two plants on sexual function in normal male mice, with the objective of finding a relatively inexpensive, widely available and effective aphrodisiac.

## Methods

### Plant material

*L. meyenii* (maca) roots collected from Yunnan, China, were identified by Professor Fa Huan Ge from Sun Yat-Sen University, and its voucher specimen number was No. 201605. *A. tuberosum* Rottl. (Chinese chive) seeds were purchased from Anguo Qide Chinese Herbal Medicine Sales Co., Ltd. (China), with a voucher specimen (No. 201612). The samples mentioned above were deposited at Nansha Research Institute, Sun Yat-Sen University, Guangzhou, China.

### Preparation of extracts

A customized supercritical fluid extractor (231–50-06) was obtained from Nantong Ruizhi Supercritical Development of Technology Co., Ltd. (China). Before the extract process, the plants were dried under 50 °C and then pulverized. 300 g of plant was placed into supercritical fluid extractor. The parameters of the supercritical fluid extraction (SFE) process were set as follows. The pressure and temperature of the extract vessel were set to 35 MPa and 50 °C, respectively, whereas the pressure and temperature of the separator were set to 8 MPa and 55 °C, respectively. CO_2_ was pumped at a constant flow rate. The extract was collected from the separator after a 1.5 h extraction. The SFE conditions for maca root extract, Chinese chive extract and the combined extracts were the same, except that extra 95% ethanol (1:1, w/v) was added as a co-solvent during the extraction for maca root. Besides, 300 g mixed plants of maca and Chinese chive (1:1, w/w, i.e. 150 g of each) were used for the extraction of combined extracts. The yield of maca root extract, Chinese chive seed extract and the combined extracts were 1.05, 18.0 and 19.05%, respectively.

### Chromatographic analysis of maca root extract, Chinese chive extract and the extract combination

HPLC analysis of maca root extract, Chinese chive seed extract and the combined extracts were performed using an Ultimate 3000 HPLC system, which consisted of an SR-3000 pump, a DAD-3000 detector and a WPS-3000 autosampler (Thermo Fisher Scientific, China). The experiment was conducted using a Kromasil 100-5C18 column (250 × 4.6 mm, 5 μm); the flow rate was set to 1.0 ml min^− 1^ of acetonitrile (solvent A) and water (0.1% phosphoric acid) (solvent B) with gradient elution (0–26 min: 85% A-15% B; 26–40 min: 85% A increasing to 95% A; 40–50 min: 95% A-5% B), and the column temperature set to 40 °C. The detection wavelength was monitored at 210 nm [31, 32].

### Animals

50 male and 100 female BALB/c mice weighing 25–30 g (9–11 weeks of age) were obtained from the laboratory animal centre of Sun Yat-Sen University. Mice were housed in stainless cages (290 cm × 185 cm) under standard environmental conditions involving a standard diet, water ad libitum, a temperature of 23 ± 2 °C, a relative humidity of 50–70%, and a day-night cycle with light from 7:00 to 19:00. The study procedures were approved by the Institutional Animal Care and Use Committee (IACUC) of Sun Yat-Sen University (IACUC-DD-17-1009).

### Male sexual behaviour

All female mice were given a subcutaneous injection of 0.02 mg of oestradiol benzoate 48 h before the start of the experiment [26]. Male mice had experienced sexual behavior training [30]. Sexually experienced male mice were divided into five groups of 10 mice. All animals were treated by oral gavage once daily for 14 consecutive days. Group 1: served as the control and received with 1 ml of tea oil (camellia); Group 2: treated with 200 mg/kg (b.w.) of maca extract; Group 3: administrated with 200 mg/kg (b.w.) of Chinese chive seed extract; Group 4: received with 200 mg/kg (b.w.) of the combination and Group 5, which served as the positive control group, received 14 mg/kg of Viagra to enhance sexual behaviour.

After 5 min for adaptation before the mating experiment, two oestrous female mice were introduced into each male mouse cage [26], and copulation was allowed. Copulatory behaviour was observed for 20 min, and four sexual behaviour parameters were recorded using a camera: mount latency (the time interval from entry into the cage to the mount of any female mouse), mount frequency, ejaculation latency (the time from first intromission to ejaculation) (characterized by longer, deeper pelvic thrusting and slow dismount followed by a period of inactivity) and ejaculation frequency (the number of ejaculations in the given time) [33].

### Serum testosterone

After male mating behaviorial test, 1 ml of blood samples were collected by removing eyeball. Serum was separated by centrifugation at 4000 rpm for 10 min at room temperature for the measurement of serum testosterone using commercial assay kits purchased from Cusabio Biotech Co., Ltd. (China).

### NO and cGMP levels in serum and penis

Penis tissue were obtained after mice sacrificed. They were sliced into pieces and homogenated. Supernatant was obtained by centrifugation at 5000 g for 5 min. NO and cGMP levels in supernatant and serum were measured through commercial assay kits purchased from Jiancheng Biological Engineering, Inc. (Nanjing, China) and Meilian Biological Technology (Shanghai, China), respectively.

### Sexual organ weight

The weight of genital organs is another index for sexual function [34]. After 14 days of treatment, the animals were sacrificed by decapitation, and the testis, seminal vesicles, preputial gland and epididymis were carefully removed and weighed. Animals’ whole-body weights were also measured.

### Statistical analysis

Statistical analyses were performed using GraphPad Prism 6.0 statistical software. Data were expressed as mean ± standard deviation (SD) and significant differences between means were calculated using one-way analysis of variance (ANOVA) followed by Dunnett’s test; differences with *P*-values less than 0.05 were regarded as significant.

## Results

### Measurement of macamides and macaene in maca root extract and the combined extracts

**Fig. 1:**
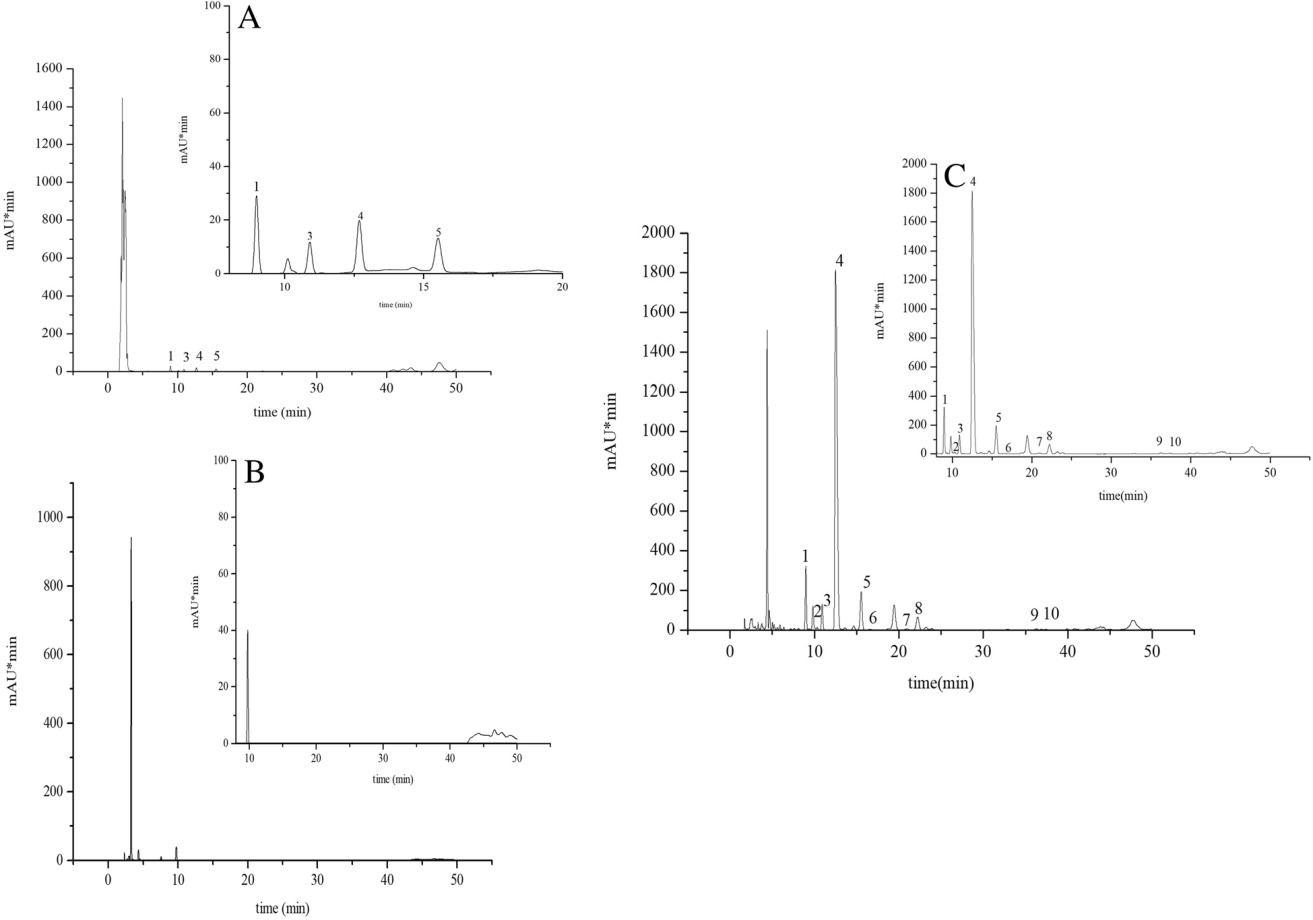
HPLC chromatographs for macamides and macaene detection of **a** maca root extract, **b** Chinese chive seed extract and **c** combined extracts from mixture of maca root and Chinese chive seed (1:1, w/w). The components labelled are: (1) (9Z,12Z,15Z)-octadecatrienoic acid; (2) (9Z,12Z,15Z)-N-(3-methoxybenzyl) octadecatrienamide; (3) (9Z,12Z,15Z)-N-benzyloctadecatrienamide; (4) (9Z,12Z)-octadecadienamide; (5) (9Z,12Z)-N-benzyloctadecadienamide; (6) N-benzylpentadecanamide; (7) N-(3-methoxybenzyl) palmitamide; (8) N-benzylpalmitamide; (9) N-(3-methoxylbenzyl) stearamide; (10) N-benzylstearamide)

We screened ten types of macamides and macaene, and their content differences between maca root extract and combined extracts were investigated. The results are shown in Fig. 1. The examined compounds were (9Z,12Z,15Z)-octadecatrienoic acid (1), (9Z,12Z,15Z)-N-(3-methoxybenzyl)octadecatrienamide (2), (9Z,12Z,15Z)-N-benzyloctadecatrienamide (3), (9Z,12Z)-octadecadienamide; (4), (9Z,12Z)-N-benzyloctadecadienamide (5), N-benzyl-pentadecanamide (6), N-(3-methoxybenzyl) palmitamide (7), N-benzylpalmitamide (8), N-(3-methoxylbenzyl) stearamide (9) and N-benzylstearamide (10). The macamides were identified via standards that were purchased from Wuhan Huashite Industrial Biotechnology Development Co., Ltd. (China). The content of these compounds in the combined extracts (202.44 mg/g) was 45-fold higher than that observed in maca root extract (4.51 mg/g, and only four of the compounds were detected). Besides, none of the compounds mentioned above were found in Chinese chive seed extract.

### Sexual behaviour study

We investigated the effects of combined extracts from maca root in combination with Chinese chive on sexual function in male mice through the course of 14 days. The results (Table 1) indicated that this combination decreased mount latency and ejaculation latency, and significantly increased ejaculation frequency (*P* < 0.05).

**Table 1:** Effects of maca extract, Chinese chive extract and combined extracts on sexual behaviour in mice (mean ± SD)

Groups	Mount latency (s)	Ejaculation latency (s)	Mount frequency (s)	Ejaculation frequency (s)
Group 1 (control)	393.90 ± 116.62	493.00 ± 92.53	4.70 ± 1.34	2.20 ± 1.14
Group 2 (maca root extract, 200 mg/kg/d, b.w.)	311.00 ± 112.85	509.70 ± 117.59	6.30 ± 1.42	2.10 ± 1.20
Group 3 (Chinese chive extract, 200 mg/kg/d, b.w.)	302.90 ± 83.08	499.40 ± 138.98	5.90 ± 1.20	2.50 ± 0.71
Group 4 (combined extracts, 200 mg/kg/d, b.w.)	282.50 ± 92.04	459.80 ± 108.39	6.60 ± 2.07*	3.50 ± 0.99*
Group 5 (Viagra, 14 mg/kg once prior to the mating experiment)	251.90 ± 71.90*	381.30 ± 92.91	7.60 ± 0.70**	3.50 ± 0.97*

*P < 0.05, **P < 0.01 vs control

### Effects of single extracts and combined extracts on serum testosterone, NO and cGMP levels in serum and penis

As shown in Table 2, the concentration of NO and cGMP were increased in all experimental groups. The levels of NO were significantly increased in Group 3 (98.94 ± 14.48, *P* < 0.05, in serum) and Group 4 (104.69 ± 8.83, *P* < 0.01, in serum; 143.18 ± 0.023, *P* < 0.05, in penis). Meanwhile, cGMP level in penis was also significantly increased in Group 4 (15.01 ± 1.89, *P* < 0.05). However, no significant differences in serum testosterone concentration were observed.

**Table 2:** Effects of maca root extract, Chinese chive extract and combined extracts on testosterone, NO and cGMP levels (mean ± SD)

Groups	Serum			Penis	
	Testosterone (nmol/L)	NO (μmol/L)	cGMP (μmol/L)	NO (μmol/L)	cGMP (μmol/L)
Group 1 (control)	15.93 ± 2.11	82.26 ± 12.19	6.17 ± 1.09	116.20 ± 0.014	12.14 ± 1.97
Group 2 (maca root extract, 200 mg/kg/d, b.w)	15.86 ± 2.06	94.25 ± 8.92	6.81 ± 1.77	118.05 ± 0.026	12.86 ± 1.04
Group 3 (Chinese chive extract, 200 mg/kg/d, b.w)	15.72 ± 4.56	98.94 ± 14.48*	7.03 ± 1.58	136.90 ± 1.81	13.21 ± 1.81
Group 4 (combined extracts, 200 mg/kg/d, b.w)	15.83 ± 2.20	104.69 ± 8.83**	7.23 ± 1.44	143.18 ± 0.023*	15.01 ± 1.89*
Group 5 (Viagra, 14 mg/kg once before mating experiment)	15.57 ± 3.30	116.62 ± 15.19*	8.26 ± 1.69*	149.73 ± 0.022*	15.39 ± 2.19*

*P < 0.05, **P < 0.01 vs control

### Effects of single extracts and combined extracts on genital indices

As shown in Table 3, the weights of sexual organs did not significantly change in any group.

**Table 3:** Effects of maca root extract, Chinese chive extract and combined extracts on genital indexes (mg/10 g) (mean ± SD)

Groups	Testis	Epididymis	Preputial gland	Seminal vesicles
Group 1 (control)	69.49 ± 7.80	12.59 ± 1.66	59.72 ± 18.88	35.65 ± 5.73
Group 2 (maca root extract, 200 mg/kg/d, b.w)	67.91 ± 10.10	12.21 ± 1.96	69.91 ± 13.49	31.82 ± 8.34
Group 3 (Chinese chive extract, 200 mg/kg/d, b.w)	68.91 ± 10.27	13.41 ± 1.56	66.39 ± 14.49	30.17 ± 6.72
Group 4 (combined extracts, 200 mg/kg/d, b.w)	68.85 ± 11.55	12.60 ± 1.57	62.40 ± 14.78	31.78 ± 6.51
Group 5 (Viagra, 14 mg/kg once before mating experiment)	64.62 ± 9.39	11.78 ± 1.25	66.32 ± 13.02	31.34 ± 6.33

## Discussion

Men’s sexual function declines over time, and erectile dysfunction (ED) brings negative effects on relationships [35], mood and psychological health [36, 37]. ED is an inability to achieve or maintain an erection sufficient for satisfactory sexual performance [38]. The maintenance of penile rigidity during erection, intromission, and ejaculation depends on blood supply [39]. Erection occurs after the activation of parasympathetic pathways prompts the release of NO from cavernous nerves and endothelial cells; the ensuing signalling pathways lead to increased cyclic guanosine monophosphate (cGMP) concentrations and decreased intracellular Ca^2+^ levels, resulting in the relaxation of penile cavernosal smooth muscle, which reduces peripheral arteriolar resistance and permits blood inflow [40, 41]. NO plays an important role in male sexual behaviour [42, 43]. In present study, we measured concentrations of serum testosterone, NO and cGMP in serum and penis to identify a potential mechanism for sextual behavior improvement of the combined extracts, and genital indices were also assessed. In Group 4, the concentrations of NO in serum and penis as well as cGMP in penis were significantly increased, which indicated the-improvement in sexual behavior caused by the combined extracts might be regulated by the NO-cGMP pathway. Testosterone, a gonadal hormone, likely enhances male sexual behaviour by increasing NO production [44, 45] and stimulating the growth of targeted tissues [46]. Unexpectedly, no significant differences in serum testosterone concentration and genital indices were observed, showing that testosterone did not participate in improving effect of the two plants extract on sexual behavior.

Research has shown that an increase in the ratio of copulating males or changes in parameters of sexual behaviour, such as a reduction in ejaculation latency, indicate improved male sexual performance [47]. The combined extracts decreased mount latency and ejaculation latency and significantly increased ejaculation frequency (*P* < 0.05). The results indicated that the combined extracts had a better efficacy in terms of sexual arousal than both single extracts. Furthermore, the chemical component analysis offered us a comprehensive understanding on active compounds in the combined extracts. The identified number and content of total macamides and macaene obviously increased in the combined extracts, because during the extraction, the extracted Chinese chive oil played a cosolvent role for supercritical CO_2_. With the help of this intermediate-produced cosolvent, the extraction efficacy was highly elevated, especially for those lipophilic ingredients like (9Z,12Z)-octadecadienamide.

## Conclusions

In summary, a novel combined extracts from maca root and Chinese chive seed produced synergistic effects with respect to improving erectile dysfunction in male mice. The mechanisms for these effects may involve the upregulation of NO and cGMP concentrations in penis.

## Data Availability

The data analyzed for this study are available from the corresponding author upon reasonable request.

## Abbreviations

cGMP: Cyclic guanosine monophosphate
ED: Erectile dysfunction
NO: Nitric oxide
